# Врожденная дисфункция коры надпочечников

**DOI:** 10.14341/probl13763

**Published:** 2026-05-20

**Authors:** М. В. Воронцова, Т. С. Кокорина, Н. Ф. Нуралиева, М. Ю. Юкина, Е. А. Трошина, Г. А. Мельниченко, Н. Г. Мокрышева

**Affiliations:** Национальный медицинский исследовательский центр эндокринологии им. академика И.И. Дедова; Московский государственный университет им. М.В. Ломоносова; Endocrinology Research Centre; Lomonosov Moscow State University; Национальный медицинский исследовательский центр эндокринологии им. академика И.И. Дедова; Endocrinology Research Centre

**Keywords:** врожденная дисфункция коры надпочечников, надпочечниковая недостаточность, 21-гидроксилаза, заместительная терапия, надпочечники, adrenal hyperplasia, congenital, adrenal insufficiency, steroid 21-hydroxylase, hormone replacement therapy, adrenal glands

## Abstract

Врожденная дисфункция коры надпочечников (ВДКН) — это группа заболеваний с аутосомно-рецессивным типом наследования, в основе которых лежит дефект ферментов стероидогенеза коры надпочечников. В зависимости от варианта ферментного блока спектр клинических проявлений ВДКН варьирует от малосимптомных до потенциально фатальных нарушений. В обзоре представлен детальный анализ шести основных форм ВДКН (липоидная гиперплазия, дефициты HSD3B2, CYP17A1, CYP21A2, CYP11B1, POR) с углубленным описанием их молекулярных основ, патогенеза и характерных клинико-лабораторных проявлений.

Особое внимание уделено современным методам генетической диагностики ВДКН, включая анализ высокогомологичного локуса CYP21A2, пренатальную и преимплантационную диагностику. Детально описаны не только современные подходы к заместительной терапии, но и перспективные методы лечения: антагонисты рецепторов кортикотропин-рилизинг-гормона, генная терапия и клеточные технологии. Уникальность работы заключается в комплексном анализе заболевания от фундаментальных основ до прикладных аспектов ведения пациентов с учетом российских клинических реалий.

## Abstract (en)

Congenital Adrenal Hyperplasia (CAH) is a group of diseases with an autosomal recessive inheritance pattern, which are caused by a defect in the enzymes involved in steroidogenesis in the adrenal cortex. Depending on the enzyme block variant, the spectrum of clinical manifestations of CAH varies from mild symptoms to potentially fatal disorders. The review provides a detailed analysis of the six main forms of CAH (lipoid hyperplasia, HSD3B2, CYP17A1, CYP21A2, CYP11B1, POR) with an in-depth description of their molecular basis, pathogenesis, and clinical and laboratory manifestations. Particular attention is paid to modern methods of genetic diagnosis of CAH, including analysis of the highly homologous CYP21A2 locus, prenatal and preimplantation diagnosis. Not only modern approaches to replacement therapy are described in detail, but also promising methods of treatment: corticotropin-releasing hormone receptor antagonists, gene- and cellbased technologies. The study's strength lies in its comprehensive analysis of the disease, spanning fundamental research to practical patient management, as applied to the realities of clinical practice in Russia.

Врожденная дисфункция коры надпочечников (ВДКН) — это группа заболеваний с аутосомно-рецессивным типом наследования, в основе которых лежит дефект одного из ферментов или транспортных белков, принимающих участие в биосинтезе кортизола в коре надпочечников. Общие звенья патогенеза данных состояний — снижение синтеза кортизола, ведущее к гиперпродукции адренокортикотропный гормон (АКТГ) и — как следствие — развитию гиперплазии коры надпочечников и накоплению метаболитов, предшествующих дефектному этапу стероидогенеза (рис. 1).

**Figure:**
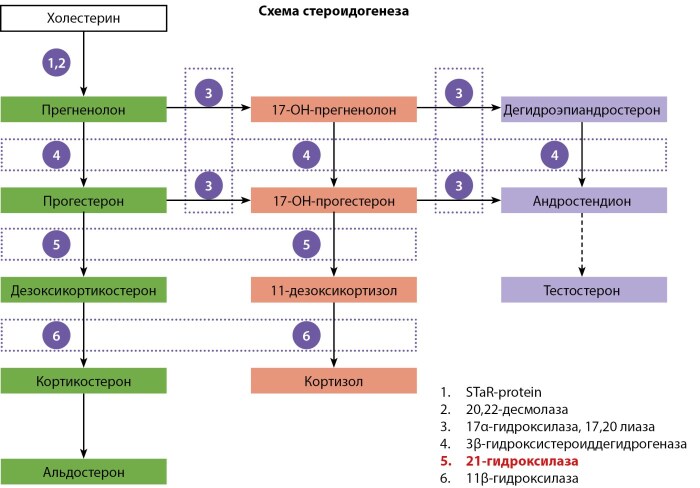
Рисунок 1. Схема стероидогенеза в коре надпочечников и расположение основных ферментов, дефект в которых может приводить к ВДКН.

Иными словами, клиническими проявлениями заболевания, в зависимости от уровня «поломки» цепочки стероидогенеза, станет комбинация явлений надпочечниковой недостаточности и нарушений строения наружных гениталий и развития ребенка. В зависимости от выраженности проявлений можно говорить и о практически фатальных, без лечения ведущих к быстрой гибели, и о медленно прогрессирующих формах.

В настоящее время описано семь форм ВДКН (дефект StAR-протеина, дефицит 20,22-десмолазы, дефицит 17α-гидроксилазы/17,20-лиазы,дефицит 3β-гидроксистероиддегидрогеназы, дефицит 21-гидроксилазы, дефицит 11β-гидроксилазы, дефицит оксидоредуктазы), шесть из которых представлены на рисунке 1, а последний фермент является донором электронов для других ферментов.

## ИСТОРИЯ ИЗУЧЕНИЯ

История изучения ВДКН восходит к середине XIX века. В 1865 г. неаполитанский врач Луиджи Де Креккио (Luigi De Crecchio) опубликовал первое подробное описание случая, при котором пациент, проживший жизнь как мужчина, при вскрытии имел женские внутренние половые органы и выраженную гиперплазию надпочечников [1][2].

В 1912 г. А. Галле (Gallais) предложил термин «адреногенитальный синдром» (adreno-genital syndrome), который широко использовался в первой половине XX в. для обозначения сочетания патологии надпочечников и нарушений полового развития [3]. В последующем в международной литературе этот термин был вытеснен более точным "congenital adrenal hyperplasia (CAH)".

Ключевой перелом в понимании патологии произошел в середине XX в. с развитием биохимии стероидов и появлением кортикостероидной терапии. В 1950 г. Уилкинс (Wilkins L.) и параллельно Бартер (Bartter F.C.) продемонстрировали, что синтетический кортизон подавляет АКТГ-зависимую гиперандрогению при врожденной гиперплазии надпочечников, заложив тем самым основы патогенетической терапии ВДКН [4–6]. К концу 1950-х годов была установлена ведущая роль дефицита 21-гидроксилазы в развитии ВДКН: клинико-биохимические работы Бонджованни и Эберлейна (Bongiovanni & Eberlein) связали клинические фенотипы (сольтеряющая и вирильная формы) с конкретным блоком стероидогенеза [7–9]. В последующие годы были описаны и другие дефициты ферментов пути биосинтеза стероидов, включая 3β-гидроксистероиддегидрогеназу и 11β-гидроксилазу, что окончательно закрепило «ферментный» принцип нозологической классификации ВДКН.

Дальнейшее развитие молекулярно-генетических исследований привело к картированию и клонированию генов 21-гидроксилазы. К 1984–1985 годам были клонированы P450c21 у млекопитающих и показано существование двух тесно расположенных копий — функционального CYP21A2 и псевдогена CYP21A1P — в составе RCCX-модуля главного комплекса гистосовместимости HLA [10][11]. Это открытие объяснило механизм частых конверсионных событий и делеций при формировании патогенных аллелей. К началу XXI в. было установлено, что около 90–95% случаев ВДКН обусловлены мутациями в гене CYP21A2 [12].

В отечественной литературе первые систематические описания заболевания относятся к 1924 г., когда О.В. Верещинский обобщил 12 случаев надпочечно-полового синдрома [13]. В 1946 г. один из основателей эндокринологии в России Н.А. Шерешевский выделил мышечный тип супрарено-генитального синдрома, соответствующий вирильной форме дефицита 21-гидроксилазы [14]. Термин «врожденная дисфункция коры надпочечников» был внедрен в отечественную практику благодаря работам Э.П. Касаткиной и ее коллег, предложивших комплексный подход к диагностике и лечению этого заболевания.

Важным этапом стало внедрение в 1977 году простого и надежного метода скрининга 21-гидроксилазной недостаточности, основанного на определении уровня 17-гидроксипрогестерона (17ОНР) радиоиммунным методом [15]. В последующие десятилетия шире интегрировали определение панели стероидов при помощи второго диагностического метода: жидкостной хроматографии с тандемной масс-спектрометрией: LC-MS/MS-панель стероидов, что улучшило позитивную прогностическую ценность теста и снизило уровень ложноположительных результатов [16].

В России федеральный неонатальный скрининг на дефицит 21-гидроксилазы был официально введен в 2006 г. (приказ Минздравсоцразвития РФ №185 от 22.03.2006), что позволило значительно улучшить раннюю диагностику и лечение этого заболевания.

## КЛАССИФИКАЦИЯ ВДКН

В настоящее время принято выделять шесть клинических вариантов ВДКН:

## СОВРЕМЕННЫЕ ПРЕДСТАВЛЕНИЯ ОБ ЭТИОЛОГИИИ И ПАТОГЕНЕЗЕ РАЗЛИЧНЫХ ФОРМ ВДКН

## Липоидная гиперплазия надпочечников

В основе большинства случаев липоидной гиперплазии надпочечников лежат дефекты гена STAR, кодирующего белок StAR (острый стероидогенный регулятор). Реже заболевание вызывают дефекты гена CYP11A1, кодирующего P450scc (20,22-десмолаза). Оба белка необходимы для осуществления первого этапа биосинтеза стероидных гормонов — превращения холестерина в прегненолон. При липоидной гиперплазии надпочечников имеет место полное нарушение синтеза всех классов стероидов как в надпочечниках, так и в половых железах.

Вследствие нарушения синтеза тестостерона в яичках уже на ранних этапах эмбриогенеза у генетических мальчиков не происходит маскулинизации наружных половых органов, при этом, однако, происходит регресс дериватов женских внутренних половых органов (нарушение формирования пола (НФП) 46XY). У девочек формирование наружных и внутренних половых органов не нарушено. Гипофункция яичников может проявиться лишь в пубертатном периоде.

Заболевание манифестирует симптомами сольтеряющего криза уже в течение первых двух недель жизни. Возможны также проявления синдрома дыхательных расстройств. В биохимическом анализе крови определяют гиперкалиемию, гипонатриемию, гипохлоремию, метаболический ацидоз, гипогликемию и повышение уровня мочевины.

Диагноз ставят на основании сочетания клиники надпочечниковой недостаточности (НН), НФП 46XY (у генетических мальчиков) и выраженного снижения секреции всех стероидных гормонов. Уровни глюкокортикоидов (ГК), минералокортикоидов (МК) и андрогенов в крови и моче (как базальные, так и после стимуляции АКТГ), как правило, не поддаются детекции. Наружные половые органы, как у генетических мальчиков, так и девочек, сформированы полностью по женскому типу. Единственный достоверный метод подтверждающей диагностики — молекулярно-генетические исследования, при которых определяют мутации в генах STAR или CYP11A1.

Лечение проводят с помощью ГК и МК (см. ниже). Генетических мальчиков целесообразно адаптировать в женском паспортном поле, и яички должны быть удалены. В пубертате пациентам обоего пола показана заместительная гормональная терапия эстрогенами.

## Дефицит HSD3B2

В основе заболевания лежат дефекты гена HSD3B2, кодирующего фермент 3β-гидроксистероиддегидрогеназу II типа. HSD3B2 необходим для превращения ∆5-стероидов прегненолона, 17-гидроксипрегненолона и ДГЭА в соответствующие им ∆4-стероиды: прогестерон, 17-гидроксипрогестерон и андростендион. HSD3B2 экспрессирован в коре надпочечников и половых железах.

Недостаточность HSD3B2 приводит к нарушению синтеза всех классов стероидов в надпочечниках и половых железах. Секретируемый в избытке ДГЭА обладает слабой андрогенной активностью, однако в периферических тканях возможен его частичный переход в андростендион и тестостерон. Суммарное количество андрогенов при этом недостаточно для адекватной маскулинизации наружных половых органов у плода с генетически мужским полом, однако избыточно для плода женского пола и приводит к незначительной вирилизации у девочек.

К типичным проявлениям заболевания относится тяжелая НН, возникающая на первом месяце жизни. Наружные половые органы как у мальчиков, так и у девочек сформированы, как правило, ближе к женскому типу.

С помощью биохимических методов исследования диагноз недостаточности HSD3B2 устанавливается на основании повышения сывороточных уровней ∆5-стероидов (прегненолон, 17-гидроксипрегненолон, ДГЭА) и повышения соотношения ∆5/∆4-стероидов. Диагноз подтверждается при выявлении мутаций в гене HSD3B2.

В большинстве случаев показана сочетанная терапия ГК и МК. Генетические мальчики в зависимости от степени маскулинизации наружных половых органов могут быть адаптированы как в мужском, так и женском паспортном поле.

## Дефицит CYP17A1

Причина заболевания — дефекты гена CYP17A1. Кодируемый данным геном фермент P450c17 активирует 17α-гидроксилирование прегненолона и прогестерона соответственно до 17-гидроксипрегненолона и 17OHP, а также последующее превращение этих стероидов в ДГЭА и андростендион посредством расщепления С17, 20-углеродного мостика. Эти ферментативные активности необходимы для биосинтеза стероидов как в надпочечниках, так и половых железах.

Результат дефицита 17α-гидроксилазы — нарушение синтеза кортизола, что приводит к гиперпродукции АКТГ и активации синтеза предшественников альдостерона. Избыточная секреция дезоксикортикостерона вызывает задержку натрия и подавление ренин-ангиотензиновой системы — основного регулятора клубочковой зоны коры надпочечников. Явных симптомов НН при нехватке данного фермента обычно не отмечают, что обусловлено избытком кортикостерона. Снижение активности 17/20лиазы ведет также к нарушению синтеза половых гормонов в надпочечниках и половых железах. В результате этого у плода с генетически мужским полом происходит недоразвитие наружных половых органов разной степени выраженности (НФП 46XY). У генетических женщин формирование внутренних и наружных половых органов не нарушено, а гипофункция яичников проявляется только в пубертате.

Наружные половые органы у генетических мальчиков при рождении имеют полное феминное строение или степень маскулинизации минимальна (НФП 46XY). Дериваты мюллеровых протоков отсутствуют. У генетических девочек внутренние и наружные половые органы при рождении не изменены. При данной форме развивается низкорениновая артериальная гипертензия.

Заболевание может быть заподозрено при выявлении НФП 46XY или первичного гипогонадизма (при женском поле) в сочетании с синдромом избытка МК. При лабораторном исследовании определяют гипокалиемию, снижение уровня половых гормонов, повышение уровня гонадотропинов, низкую активность ренина в плазме и повышение концентраций кортикостерона и дезоксикортикостерона. Диагноз подтверждается при выявлении мутаций в гене CYP17A1.

Необходима заместительная терапия ГК, направленная на возмещение дефицита кортизола и подавление АКТГ-зависимого синтеза дезоксикортикостерона. Генетических мальчиков целесообразно адаптировать в женском паспортном поле. Начиная с пубертатного возраста проводят заместительную терапию эстрогенами.

## Дефицит CYP21A2

Более 90% всех случаев ВДКН приходится на дефицит CYP21A2. Популяционная частота классического варианта данного заболевания варьирует в разных странах от 1 на 10 000 до 1 на 15 000, достигая в отдельных генетических изолятах частоты 1 на 280 (эскимосы Юпик) и 1 на 2000 (остров Реюнион в Индийском океане), в России — 1:100.

В основе заболевания лежат мутации гена CYP21A2, кодирующего 21-гидроксилазу. CYP21A2 отвечает за 21-гидроксилирование прогестерона и 17OHP, соответственно, в дезоксикортикостерон и 11-дезоксикортизол.

При дефиците CYP21A2 нарушен синтез ГК и МК в коре надпочечников. Дефицит кортизола приводит к АКТГ-опосредованному повышению активности интактных этапов стероидогенеза и — как следствие — избыточной продукции субстанций, для синтеза которых не нужно 21-гидроксилирование, преимущественно 17OHP — субстрата для синтеза андростендиона и тестостерона. Избыточная секреция андрогенов надпочечниками плода с генетически женским полом ведет к вирилизации наружных половых органов, степень которой может варьировать от умеренно выраженной клиторомегалии (стадия I по Прадеру) до полного сращения больших половых губ и формирования пенильной уретры (стадия V по Прадеру). Внутренние половые органы у девочек при этом не изменены. У мальчиков внутриутробный избыток надпочечниковых андрогенов не оказывает заметного эффекта на формирование половых органов.

В зависимости от выраженности дефицита CYP21A2 клинически выделяют сольтеряющую, простую вирильную и неклассическую формы заболевания.

При рождении у девочек при недостаточности CYP21A2 отмечают различную степень вирилизации наружных половых органов. В последующем избыток надпочечниковых андрогенов как у мальчиков, так и у девочек приводит к развитию синдрома ложного ППС. При сольтеряющей форме, помимо указанных выше симптомов, заболевание проявляется также НН. На фоне сольтеряющего криза в биохимическом анализе крови определяют гиперкалиемию, гипонатриемию, гипохлоремию, метаболический ацидоз, гипогликемию и повышение уровня мочевины.

С помощью биохимических методов исследования диагноз устанавливают при обнаружении повышенного уровня 17OHP в крови. К типичным изменениям гормонального профиля относится также повышение активности ренина в плазме. Подтверждающая диагностика осуществляется при выявлении мутаций в гене CYP21A2. В настоящее время проводится неонатальный скрининг на дефицит CYP21A2. Скрининг основан на определении повышенного уровня 17OHP в пятне крови, полученном на 4‑е сутки жизни (у недоношенных — на 10‑е сутки).

## Дефицит CYP11B1

Причина заболевания — дефекты гена CYP11B1, кодирующего фермент 11β-гидроксилазу. CYP11B1 необходим для 11β-гидроксилирования 11-дезоксикортикостерона в кортикостерон и 11-дезоксикортизола в кортизол.

При дефиците CYP11B1 нарушен синтез кортизола, что приводит к стимуляции секреции АКТГ и избыточной продукции предшественников, образующихся проксимально к данному этапу стероидогенеза. Избыток дезоксикортикостерона, обладающего минералокортикоидной активностью, ведет к задержке натрия, повышению артериального давления и подавлению активности ренин-ангиотензиновой системы. Кроме этого, при недостаточности CYP11B1 происходит накопление предшественников андрогенов, что служит причиной развития вирилизации и преждевременного полового созревания надпочечникового генеза.

При недостаточности CYP11B1 у девочек при рождении отмечают вирилизацию наружных половых органов. Степень вирилизации может быть столь значительной (стадии IV–V по Прадеру), что пациентов ошибочно регистрируют в мужском поле и воспитывают как мальчиков. У большинства больных заболевание сопровождается также повышением артериального давления. Следует отметить, что, хотя для данного варианта ВДКН характерен избыток МК, иногда у детей раннего возраста заболевание может проявляться синдромом потери соли.

Биохимически диагноз подтверждается при выявлении повышенных уровней 11-дезоксикортизола и дезоксикортикостерона в крови. Активность ренина в плазме снижена. Диагноз верифицируется на молекулярном уровне при исследовании гена CYP11B1.

При дефиците CYP11B1 необходима заместительная терапия ГК, направленная на возмещение дефицита кортизола и подавление АКТГ-зависимого синтеза дезоксикортикостерона и предшественников надпочечниковых андрогенов. Так же, как и при дефиците CYP21A2, у девочек проводят феминизирующую пластику наружных половых органов.

## Дефицит POR

Причина заболевания — дефекты гена POR, кодирующего фермент P450-оксидоредуктазу, которая представляет собой флавопротеин, снабжающий молекулами кислорода все микросомальные ферменты семейства цитохрома P450, включая CYP21A2 и CYP17A1.

При недостаточности P450-оксидоредуктазы у девочек при рождении можно наблюдать умеренную вирилизацию наружных половых органов (как правило, не превышающую стадию III по Прадеру). Постнатального прогрессирования вирилизации нет. У мальчиков формирование наружных половых органов либо не нарушено, либо наблюдается клиника умеренно выраженного НФП 46XY, но с преобладанием мужского строения. У части пациентов отмечаются умеренно выраженные проявления сочетанного дефицита ГК и МК.

Особенностью заболевания является возможность вирилизации матери при беременности плодом с дефицитом POR вследствие нарушения работы ферментов ароматизации дегидроэпиандростерона (ДЭА) в плаценте.

Биохимически диагноз может быть заподозрен при выявлении повышенных уровней 17OHP и 17-гидроксипрегненолона. Диагноз верифицируется на молекулярном уровне при исследовании гена POR.

При дефиците P450-оксидоредуктазы некоторым больным показана заместительная терапия ГК и МК. Потребность в лечении устанавливают индивидуально, исходя из клинических проявлений, показателей АКТГ и активности ренина в плазме. При необходимости проводят корригирующую пластику наружных половых органов.

## Неклассическая ВДКН

Неклассическая форма дефицита 21-гидроксилазы (нВДКН) не сопровождается признаками НН и проявляется поздно — обычно после пубертатного возраста. У мужчин это заболевание практически никогда не диагностируется и не требует лечения в связи с отсутствием характерных признаков. У женщин отмечаются признаки умеренной гиперандрогении, нарушение менструального цикла и проблемы с вынашиванием беременности [17–19].

## ПРОБЛЕМЫ ДИАГНОСТИКИ И ЛЕЧЕНИЯ ВДКН

## Пренатальная диагностика

Пренатальная диагностика ВДКН рекомендована в тех случаях, когда оба родителя являются носителями патогенных вариантов гена CYP21A2, что чаще всего выявляется после рождения в семье ребенка с ВДКН. Исторически применявшиеся биохимические подходы (определение стероидов в амниотической жидкости) и HLA-типирование сегодня уступили место прямому генетическому анализу клеточного материала плода [20]. Инвазивная диагностика включает биопсию хориона (на 10–12‑й неделе гестации) и амниоцентез (чаще 15–16-я неделя) с тестированием «семейных» вариантов CYP21A2 и оценкой перестроек в RCCX-локусе (например, MLPA для делеции/дупликаций и химерных вариантов).

Перспективным направлением является поиск неинвазивных методов диагностики ВДКН на пренатальном этапе, наиболее существенный прогресс связан с определением внеклеточной фетальной ДНК в плазме матери. Внеклеточная фетальная ДНК (cffDNA) — это короткие фрагменты ДНК трофобластического происхождения, циркулирующие в плазме беременной с ранних сроков и быстро элиминирующиеся после родов [21]. Методика пренатального определения пола плода заключается в детекции SRY посредством ПЦР амплификации внеклеточной фетальной ДНК и может применяться на самых ранних сроках беременности (с 6–8 недель). Метаанализ 90 исследований (9965 беременностей; 10 587 тестов) продемонстрировал высокую диагностическую точность определения пола плода по cffDNA с чувствительностью 96,6% и специфичностью 98,9% вне зависимости от триместра/недели тестирования (начиная ≥5 нед. гестации) [22]. Раннее определение пола плода позволит минимизировать потенциально неоправданную пренатальную терапию дексаметазоном плодов мужского пола.

Выявление патогенных вариантов в гене CYP21A2, ассоциированных с ВДКН, сопряжено с методологическими сложностями, обусловленными особенностями его геномного локуса. Высокая гомология между функциональным геном CYP21A2 и его псевдогеном CYP21A1P в регионе RCCX комплекса HLA (6p21.3), составляющая ~98% в кодирующих областях и ~96% в интронах, затрудняет интерпретацию данных короткоридного секвенирования без предварительного генно-специфичного обогащения и последующей биоинформатической фильтрации ридов, происходящих из псевдогена. В связи с этим рутинные диагностические протоколы комбинируют таргетное секвенирование CYP21A2 с анализом крупных перестроек методом MLPA, а для разрешения сложных случаев привлекают технологии длинного прочтения (long-read sequencing) [12][23].

Спектр патогенных аллелей при 21-гидроксилазной недостаточности формируется преимущественно за счет двух механизмов: микроконверсии последовательностей от CYP21A1P к CYP21A2 (около 70–75% случаев) и крупных делеций или химерных перестроек (около 20–30%), в то время как на долю иных вариантов, включая de novo мутации, приходится незначительный процент [24][25].

Попытки использовать ПЦР-амплификацию для специфического увеличения только CYP21A2 также сопряжены с трудностями. Высокая гомология затрудняет дизайн праймеров, которые были бы абсолютно специфичны к функциональному гену и не гибридизировались бы с псевдогеном. Даже незначительная неспецифическая амплификация CYP21A1P приведет к ложноположительным или ложноотрицательным результатам, поскольку ампликоны будут содержать смесь последовательностей от обоих локусов.

Проблема высокой гомологии усугубляется в контексте неинвазивной пренатальной диагностики на основе внеклеточной фетальной ДНК плода. Доля фетальной ДНК в материнской плазме вариабельна, и в ранние сроки беременности обычно составляет ~3–10% от общей циркулирующей ДНК, достигая ~10–15% к 10–20‑й неделе гестации [26]. Кроме того, фетальная ДНК фрагментирована. Прямое секвенирование такой сложной смеси без предварительного обогащения гаплотипов приведет к тому, что сигнал от потенциально мутантного фетального аллеля CYP21A2 будет «зашумлен» сигналом от многочисленных копий псевдогена CYP21A1P, присутствующих в материнской ДНК и, возможно, в ДНК самого плода.

Для преодоления проблемы гомологии применяются высокоточные непрямые методы, такие как гаплотипориентированная диагностика (RHDO). Вместо поиска самой мутации этот подход анализирует уникальные фланкирующие SNP-маркеры, которые однозначно идентифицируют родительские гаплотипы, несущие дефектный ген. Таким образом, можно установить, унаследовал ли плод «рисковый» гаплотип, определив аллельное происхождение фетальной ДНК на основе анализа ДНК родителей и, если доступен, пробанда (ранее рожденного ребенка с заболеванием) [26–29].

Клинические исследования подтверждают высокую точность метода при его выполнении в специализированных центрах, в том числе на ранних сроках беременности (с 5–6 недель) [29]. Для успешной диагностики необходимо соблюдение нескольких критически важных условий: достаточная фракция фетальной ДНК, применение CYP21A2-специфичного таргетного обогащения и биоинформатической фильтрации данных для исключения чтений псевдогена CYP21A1P. Кроме того, обязателен контроль рекомбинаций в участке с высокой гомологией, для чего используются маркеры по обе стороны гена, позволяющие детектировать кроссинговер. Также следует учитывать, что в случае близкородственных браков информативность метода снижается.

## Преимплантационная диагностика

Преимплантационная генетическая диагностика (ПГД) моногенных заболеваний является эффективной стратегией профилактики рождения ребенка с дефицитом 21-гидроксилазы в парах-носителях CYP21A2 и выполняется в рамках ЭКО с селективным переносом эмбрионов, не унаследовавших заболевание [30].

Современные протоколы ПГД рекомендуют проведение биопсии на стадии бластоцисты (5–6‑й день развития), когда эмбрион состоит приблизительно из 120 клеток, а трофэктодерма четко дифференцирована от внутренней клеточной массы. Забор 5–10 клеток трофэктодермы обеспечивает достаточный материал для генетического анализа при минимальном воздействии на эмбриональные структуры, ответственные за дальнейшее развитие плода [31][32]. Альтернативный подход — биопсия полярных тел ооцита — позволяет проводить преконцепционную диагностику, однако при аутосомно-рецессивных заболеваниях, таких как ВДКН, этот метод обладает принципиальным ограничением, поскольку не обеспечивает информации об отцовском аллеле [31].

## Диагностика классических форм дефицита 21-гидроксилазы

В качестве основного метода выявления классических форм дефицита 21-гидрокислазы рекомендуется проводить неонатальный скрининг.

С середины 2006 г. неонатальный скрининг (исследование 17 оксипрогестерона) у новорожденных на 4‑й день жизни, у недоношенных — на 7–10‑й день жизни) был внедрен в России, что позволяет поставить диагноз и начать лечение еще в раннем детском возрасте. Поэтому диагностика классических форм во взрослом возрасте обычно уже не требуется. Однако иногда заболевание не диагностируется вовремя, и установление диагноза требуется в старшем возрасте. Обычно эта ситуация возникает у взрослых при сочетании несоответствия паспортного и генетического пола или при длительно существующей тяжелой вирилизации.

В случаях необходимости постановки диагноза в более позднем возрасте рекомендуется использовать как главный диагностический маркер уровень 17ОНР в сыворотке крови в ранние утренние часы.

Диагностика дефицита 21-гидроксилазы основана на определении уровня 17ОНР — предшественника кортизола, находящегося непосредственно над ферментативным блоком. При классических формах его уровень обычно значительно превышен — более 300 нмоль/л или более 100 нг/мл. Кроме того, отмечается значительное повышение уровней тестостерона, андростендиона и других предшественников половых стероидов. При таких показателях диагноз не вызывает сомнений, дополнительного подтверждения не требуется. Для окончательного уточнения диагноза и с целью генетического консультирования пациентов используется генетическое исследование на наличие мутаций в гене 21-гидроксилазы — CYP21A2.

## Диагностика неклассической формы дефицита 21-гидроксилазы

Диагностику нВДКН рекомендуется проводить у женщин с признаками гирсутизма, алопеции, акне, нарушениями менструального цикла, бесплодием и/или привычным невынашиванием беременности.

Рекомендуется проводить диагностику нВДКН по результатам утреннего уровня 17ОНР в сыворотке крови в раннюю фолликулярную фазу, далее при необходимости диагноз можно подтвердить с помощью стимулирующего теста с тетракозактидом (в РФ в настоящее время не зарегистрирован) [33][34].

Не рекомендуется исследовать для диагностики нВДКН уровни дигидротестостерона, андростендиола глюкоронида, 17-кетостероидов мочи [35].

Забор крови на 17ОНР проводят рано утром в фолликулярную фазу цикла (не позднее 5–7 дня), при аменорее — в любой день, строго вне беременности. Нормой считаются показатели менее 6 нмоль/л или менее 2 нг/мл, ниже этих уровней нВДКН практически не встречается. Следует помнить, что референсные значения, которые приводятся различными лабораториями, обычно отличаются и могут быть значительно ниже указанных «отрезных точек» для диагностики нВДКН. В случае значений базального 17ОНР более 30 нмоль/л или 10 нг/мл, диагноз ВДКН считается подтвержденным, и дополнительной диагностики не требуется. При пограничных значениях 17ОНР (6–30 нмоль/л или 2–10 нг/мл — так называемая серая зона), выявленных минимум при двукратном определении, необходимо проводить дополнительный стимулирующий тест с тетракозактидом — синтетическим аналогом АКТГ, что является золотым стандартом диагностики ВДКН во всем мире (табл. 1, 2) [33][34][36][37].

При положительных или сомнительных результатах определения 17ОНР или теста с тетракозактидом, а также в целях генетического консультирования далее рекомендуется проводить генотипирование в сертифицированной лаборатории.

**Table:** Таблица 1. Диагностика ВДКН вследствие дефицита 21-гидроксилазы

Базальный уровень 17ОНР
<6 нмоль/л(<2 нг/мл)	6–30 нмоль/л(2–10 нг/мл)	>30 нмоль/л(>10 нг/мл)
Дополнительная диагностика не требуется	Показано проведение пробы с тетракозактидом	Дополнительная диагностика не требуется
	<30 нмоль/л(<10 нг/мл)	>30 нмоль/л(>10 нг/мл)	
Патологии не выявлено	Неклассическая ВДКН

**Table:** Таблица 2. Протокол проведения пробы с тетракозактидом-депо

Протокол проведения пробы с тетракозактидом-депо:
- исходно исследуется базальный уровень 17ОНР утром в раннюю фолликулярную фазу цикла;- после забора крови глубоко внутримышечно вводится 1 мг тетракозактида-депо;- через 12 и/или 24 часа исследуются уровни 17ОНР и кортизола.

ВДКН является аутосомно-рецессивным заболеванием, поэтому для подтверждения диагноза необходимо, чтобы было выявлено одновременно 2 мутации в определяемых положениях гена (это может быть гомозиготная мутация либо 2 разные мутации в гетерозиготном положении). При выявлении лишь одной гетерозиготной мутации человек считается здоровым гетерозиготным носителем, и лечения не требуется.

## Диагностика дефицита 11β-гидроксилазы

Диагностику дефицита 11β-гидроксилазы рекомендуется проводить по уровню 11-дезоксикортизола в сыворотке крови у пациентов с нетипичным течением ВДКН или появлением артериальной гипертензии.

В рутинной практике это возможно сделать только в ходе проведения мультистероидного анализа. При подозрении на гипертоническую форму ВДКН: при низком уровне калия и активности ренина плазмы вне приема МК или передозировки ГК, при повышении артериального давления у пациентов с ранее выявленной вирильной формой заболевания, а также при отсутствии типичных мутаций в гене CYP21 пациента необходимо направить в специализированные центры для уточнения диагноза и подбора терапии.

## Инструментальная диагностика при классических формах дефицита 21-гидроксилазы и 11β-гидроксилазы

Рекомендуется использовать такие методы инструментальной диагностики, как УЗИ, КТ, денситометрию, эзофагогастродуоденоскопию (ЭГДС) с целью оценки осложнений заболевания и длительной заместительной терапии глюкокортикостероидами.

Инструментальная диагностика у взрослых пациентов направлена на выявление вторичных образований в надпочечниках, особенно при эпизодах длительной декомпенсации в анамнезе (УЗИ, КТ надпочечников). Необходимый этап — оценка состояния репродуктивной системы, используемые скрининговые методики — УЗИ органов малого таза у женщин (выявление поликистоза яичников), тестикул у мужчин (диагностика образований яичек из остаточной надпочечниковой ткани (TART)). При первичном обращении пациента проводится инструментальная оценка состояния минеральной плотности костной ткани (проведение денситометрии минимум 2 отделов — позвоночника, проксимального отдела бедренной кости). Необходимо помнить о важности мониторирования состояния желудочно-кишечного тракта пациентов — проведение УЗИ органов брюшной полости, ЭГДС, т.к. пациенты находятся на пожизненной терапии глюкокортикостероидами.

## Редактирование генома эмбрионов

Потенциальной профилактикой тяжелых наследственных заболеваний с ограниченными возможностями терапии, таких как врожденная гиперплазия коры надпочечников (ВДКН), может стать редактирование генома эмбрионов. Технологии программируемых нуклеаз, в частности CRISPR/Cas9, теоретически позволяют осуществлять коррекцию патогенных вариантов уже на стадии зиготы, что открывает перспективу полного устранения заболевания у будущего потомства [38][39].

Научные исследования демонстрируют принципиальную осуществимость данного подхода, включая работы по изучению раннего эмбриогенеза человека. Однако на пути клинической реализации сохраняются существенные методологические сложности. Ключевыми проблемами являются мозаицизм редактирования, временная динамика активности нуклеаз и сложности предимплантационного контроля результатов [38–40]. Международное научное сообщество проявляет осторожный подход к вопросам клинического применения наследуемого редактирования генома. Ведущие экспертные организации, включая ВОЗ, акцентируют внимание на необходимости поэтапного и прозрачного развития исследований в этой области при обеспечении строгого этического контроля. Особое значение придается вопросам безопасности, определению критериев допустимости вмешательства и созданию эффективных механизмов общественного обсуждения [41].

В контексте ВДКН редактирование генома эмбрионов в настоящее время остается преимущественно предметом фундаментальных исследований. Существующие клинические подходы, такие как ПГД и пренатальная диагностика, продолжают оставаться основными методами профилактики заболевания, в то время как технологии геномного редактирования требуют дальнейшего изучения.

## Генетические исследования

При точечных мутациях и сохранении до 50% активности фермента развивается неклассическая форма ВДКН. В случае гетерозиготных мутаций клиническая картина определяется более «легкой» мутацией (табл. 3) [37].

**Table:** Таблица 3. Частотное распределение различных генотипов и генетически-фенотипические корреляции у взрослых пациентов с дефицитом 21-гидроксилазы в РФ

Мутация	Процент встречаемости	Форма заболевания (%)
I2spl/I2spl	47%	Сольтеряющая 85%Вирильная 15%
I172N/I172N	33%	Сольтеряющая 21%Вирильная 78%
E3del/E3del	5%	Сольтеряющая 100%
R356W/R356	5%	Сольтеряющая 100%
Q318X/Q318/X	2%	Сольтеряющая
I2spl/I172N	2%	Сольтеряющая
I2spl/P30L	2%	Вирильная
I2spl/V281L	2%	Вирильная
I172N/R356W	2%	Вирильная

Особенности строения гена CYP21A2, а именно наличие в непосредственной близости псевдогена CYP21A1, приводят к тому, что примерно за 90% случаев ВДКН отвечают 12 наиболее частых патогенных вариантов, образовавшихся в результате рекомбинации генов. С целью удешевления и ускорения исследования разработаны методики аллель-специфической ПЦР для определения наиболее частых мутаций. Однако этот метод имеет ряд ограничений: он не позволяет выявить спорадические патогенные варианты. С помощью него невозможно различить гомо- и гемизиготные варианты, что может играть принципиальное значение при генетическом консультировании при планировании беременности. Более точным и предпочтительным методом генетической диагностики является секвенирование гена CYP21A2, т.к. оно позволяет определить спорадические патогенные варианты. Так, при неклассических формах ВДКН возможна идентификация как патогенных вариантов, приводящих к незначительной потере функции белка, так и вариантов, ответственных за полную инактивацию белка.

Однако, как и аллель-специфическая ПЦР, при выявлении двух и более мутаций секвенирование не позволяет определить, находятся ли они в компаунд-гетерозиготе или в одном положении гена. Кроме того, сложность в диагностике представляет определение количества копий гена, больших делеций и перестроек. При подозрении на подобные случаи можно использовать количественную ПЦР в реальном времени или мультиплексную лигазозависимую амплификацию (MLPA). В связи с вышеперечисленными сложностями, несоответствие лабораторных, клинических и генетических параметров не может являться показанием к снятию диагноза ВДКН. В таких спорных случаях большинство исследователей рекомендуют проводить диагностику с использованием нескольких молекулярно-генетических методов, а также проводить генетическое исследование родителей пациентов [33][37][42–49].

## ПРИНЦИПЫ ЗАМЕСТИТЕЛЬНОЙ ТЕРАПИИ ВРОЖДЕННОЙ ДИСФУНКЦИИ КОРЫ НАДПОЧЕЧНИКОВ

## Заместительная терапия глюкокортикоидами

При классических формах дефицита 21-гидроксилазы (вирильной, сольтеряющей), которые проявляются дефицитом кортизола, показана терапия ГК. Основные цели лечения:

1) подавить избыточный синтез надпочечниковых андрогенов;

2) подобрать режим и дозы ГК так, чтобы они максимально соответствовали физиологическому ритму кортизола и при этом значительно не нарушали качество жизни пациента (удовлетворительное общее самочувствие, способность вести свою обычную профессиональную деятельность и максимально приближенный к обычному образ жизни);

3) избежать развития адреналового криза;

4) избежать хронической передозировки и ее отдаленных нежелательных эффектов (остеопороз, повышение кардиоваскулярных рисков, метаболический синдром).

Наиболее физиологичным препаратом для терапии дефицита ГК при ВДКН является таблетированный гидрокортизон. Гидрокортизон обладает как глюкокортикоидным, так и минералокортикоидным действием. При терапии гидрокортизоном определенные сложности возникают из-за относительно короткого периода действия препарата (табл. 4). При двухразовом приеме достаточно типичны жалобы пациентов на слабость в вечерние часы и рано утром до приема таблеток [50].

**Table:** Таблица 4. Лекарственные препараты для лечения ВДКН, фармакокинетика

Названия препарата	Продолжительность действия	Период полувыведения	Период полураспада
Гидрокортизон	короткая	90 минут	6–12 часов
Преднизолон, метилпреднизолон	средняя	200 минут	12–36 часов
Дексаметазон, триамциналон	длительная	250 минут	36–48 часов

Отрицательным свойством синтетических препаратов (преднизолон, дексаметазон) является их относительно узкий терапевтический диапазон. С особой осторожностью следует назначать препараты длительного действия (особенно дексаметазон), при использовании которых высок риск передозировки и ее последствий.

В отличие от активных ГК гидрокортизона и преднизолона, для активации кортизона ацетата требуется фермент печени 11-гидроксистероиддегидрогеназа 1 типа. Поэтому заместительная терапия кортизона ацетатом может приводить к значительной фармакокинетической вариабельности и не рекомендуется при ВДКН [51].

Препаратом выбора у детей и подростков является исключительно гидрокортизон, поскольку при лечении синтетическими препаратами отмечается задержка роста [52].

Согласно последним результатам исследований, физиологическая суточная секреция кортизола составляет 5–8 мг/м², что существенно ниже, чем считали ранее — 10–12 мг/м². Таким образом, с учетом метаболизма препарата гидрокортизона, эквивалентная суточная доза должна составлять 15–25 мг. В отдельных случаях возможно увеличение дозы препарата.

Фармакокинетическое исследование показало, что уровень гидрокортизона резко повышается через 1–2 часа после приема и быстро снижается до очень низких значений через 5–7 часов. Чтобы воспроизвести циркадный ритм, ГК обычно назначаются в два или три приема, в редких случаях — в четыре приема, приблизительно через каждые 5–6 часов с перераспределением самой высокой дозы (приблизительно ½ или 2/3) на утро, сразу после пробуждения, и самой низкой в последний прием. Прием последней дозы препарата должен назначаться не позднее, чем за 4–6 часов до сна, чтобы не спровоцировать бессонницу и нарушение чувствительности к инсулину. Согласно результатам многочисленных исследований, 3- и 4-кратные режимы дозирования более физиологичны и предпочтительны для заместительной терапии глюкокортикоидной недостаточности, однако менее частое дозирование может ассоциироваться с лучшей комплаентностью пациентов. Как альтернатива гидрокортизону, особенно в тех случаях, когда не удается достичь супрессии утренней гиперсекреции андрогенов или у пациента нет улучшения с точки зрения качества жизни и работоспособности, или пациенту тяжело придерживаться многократного режима дозирования, возможно назначение преднизолона (5–7,5 мг/сутки), перорально дважды в день, дексаметазона в дозе 0,25–0,5 мг один раз в день или метилпреднизолона (4–6 мг/сутки) дважды в день [34]. Нередко с целью супрессии гиперсекреции надпочечниковых андрогенов пациентам с ВДКН назначаются супрафизиологические дозы ГК, что может приводить к развитию ятрогенного гиперкортицизма.

К сожалению, надежные лабораторные критерии адекватности заместительной (т.е. направленной на достижение компенсации глюкокортикоидной недостаточности) терапии ВДКН глюкокортикоидами на сегодняшний день отсутствуют, и подбор терапии базируется практически исключительно на данных клинической картины и опыте врача. Исследование уровня кортизола в крови на фоне лечения не целесообразно, однако может применяться, чтобы адекватно подобрать дозу, если подозревается синдром мальабсорбции или нарушение метаболизма кортизола, например, при приеме лекарственных средств, которые влияют на период его полураспада. Измерение АКТГ плазмы для контроля за терапией не рекомендуется, так как в норме в течение суток фиксируются значительные колебания уровня гормона, который может повышаться и при адекватном лечении, особенно при приеме ГК короткого действия.

Можно выделить следующие критерии адекватности глюкокортикоидной терапии ВДКН (в отношении компенсации глюкокортикоидной недостаточности):

Однако для оценки адекватности супрессии секреции надпочечниковых андрогенов проводится исследование следующих показателей: андростендион, 17ОНР и тестостерон. Недавно обнаружено, что такие метаболиты, как 21-дезоксикортизол и 11-оксистероиды, могут более точно отражать продукцию андрогенов при ВДКН. Стероиды могут быть определены в крови, слюне, моче или высушенных образцах крови на фильтровальной бумаге. При этом золотым стандартом для измерения содержания гормонов в крови и слюне является ЖХ-МС/МС, в то время как для определения гормонов в моче рекомендуется газовая хроматография-масс-спектрометрия. При оценке результатов исследований необходимо учитывать циркадный ритм секреции, а также время приема ГК. При этом супрессия уровня 17ОНР не является целью лечения, а напротив, свидетельствует о передозировке. Интерпретация уровня андростендиона должна осуществляться с учетом пола и возраста пациента.

При адекватном лечении у пациентов с ВДКН фиксируются уровни 17ОНР и андростендиона на верхней границе или немного выше верхней границы референсного интервала. Однако клиницисты не должны ориентироваться исключительно на результаты лабораторных исследований, необходимо в первую очередь оценивать клиническую картину заболевания, в частности регулярность менструального цикла и симптомы избытка андрогенов у женщин [34].

С целью профилактики аддисонического криза (острый гипокортицизм; острая НН — жизнеугрожающее осложнение классических форм ВДКН, возникающее при несоответствии уровня кортизола увеличенной потребности в нем) рекомендуется корректировать дозу ГК в определенных условиях (табл. 5) [53].

**Table:** Таблица 5. Коррекция заместительной терапии при первичной надпочечниковой недостаточности [72–75]

Условие	Действие
Госпитализация на усмотрение лечащего врача
Сильный эмоциональный стресс	Кратковременный: коррекция не требуется (возможен дополнительный прием 10 мг гидрокортизона за 1 час до стрессовой ситуации)Длительный выраженный стресс: увеличение суточной дозы гидрокортизона на 10–20 мг
Сменная работа	Адаптация дозы глюкокортикоидов в соответствии с режимом сна и бодрствования
Лихорадка, нетяжелые травмы, умеренная боль	Увеличение дозы гидрокортизона в 2 раза при t 38 °С, в 3 раза — при t 39 °С до нормализации (обычно 2–3 дня); повышенное употребление электролитсодержащих жидкостей. Возвращение к исходной терапии после выздоровления: в течение 1–2 дней, если доза удваивалась, и в течение 2 дней, если доза утраивалась
Гастроэнтерит, пищевая токсикоинфекция (особенно при рвоте и поносах) или травма, сильная боль	Раствор гидрокортизона (в виде гидрокортизона сукцината натрия) внутримышечно 100 мг в сутки (например, утром 50 мг, днем 25 мг и вечером 25 мг) до полного выздоровления; вернуться к базисной заместительной терапии в течение 1–2 дней при отсутствии осложнений
Стоматологические процедуры	Увеличение дозы гидрокортизона в 2 раза за 2 часа до проведения стоматологических процедур длительностью менее часа под местной анестезией. Со следующего дня — возвращение к прежней схеме лечения при отсутствии осложнений.Увеличение дозы гидрокортизона в 2 раза или внутривенное или внутримышечное введение 25–50 мг раствора гидрокортизона (в виде гидрокортизона сукцината натрия) за 2 часа до проведения стоматологических процедур длительностью более часа под местной анестезией. Со следующего дня при отсутствии осложнений — возвращение к прежней схеме лечения, если доза не увеличивалась больше чем в 2 раза. В противном случае — снижение до стандартной дозы постепенно в течение 2–3 дней
Малоинвазивные несложные вмешательства (например, эзофагогастродуоденоскопия)	Внутримышечное введение раствора гидрокортизона (в виде гидрокортизона сукцината натрия) в дозе 25–50 мг в сутки (например, 25 мг до вмешательства и, при необходимости, 25 мг после вмешательства) с последующим переходом к привычной схеме лечения на следующий день после вмешательства
Любая жизнеугрожающая ситуация до госпитализации	Внутривенное болюсное (или внутримышечное) введение раствора гидрокортизона (в виде гидрокортизона сукцината натрия) в дозе 100 мг
Госпитализация обязательна
Артериография (коронарография) и другие сложные малоинвазивные вмешательства	Непосредственно перед процедурой — внутривенное или внутримышечное введение 100 мг раствора гидрокортизона (в виде гидрокортизона сукцината натрия). После исследования — в течение суток таблетированный гидрокортизон в удвоенной дозе с последующим переходом к привычной схеме лечения
Подготовка к колоноскопии	Перед началом подготовки — внутривенное или внутримышечное введение 25 мг раствора гидрокортизона (в виде гидрокортизона сукцината натрия). Непосредственно перед и после процедуры — внутривенное или внутримышечное введение 25 мг раствора гидрокортизона (в виде гидрокортизона сукцината натрия). После исследования — в течение суток таблетированный гидрокортизон в удвоенной дозе с последующим переходом к привычной схеме лечения
Тяжелые инфекции (пневмония, пиелонефрит)	Раствор гидрокортизона (в виде гидрокортизона сукцината натрия) внутривенно 25 мг каждые 8 часов до полного выздоровления; вернуться к базисной заместительной терапии в течение 1–2 дней при отсутствии осложнений
Госпитализация обязательна
Хирургическое лечение (несложное)	Внутривенное введение раствора гидрокортизона (в виде гидрокортизона сукцината натрия) в дозе 75 мг в сутки (например, 25 мг каждые 8 часов); вернуться к базисной заместительной терапии в течение 1–2 дней при отсутствии осложнений
Большое хирургическое вмешательство под общим наркозом, роды (кесарево сечение)	Внутривенное введение раствора гидрокортизона (в виде гидрокортизона сукцината натрия) в дозе 100 мг болюсно (непосредственно до операции/в начале активных родов (расширение шейки матки на 4 см и/или схватки каждые 5 минут в течение часа)), далее непрерывное введение 200 мг в сутки (или по 50 мг каждые 6 ч внутривенно или внутримышечно); непрерывное внутривенное введение жидкостей (5%-ный раствор декстрозы и 0,20%-ный или 0,45%-ный раствор натрия хлорида).1‑й день после операции — внутримышечное введение раствора гидрокортизона (в виде гидрокортизона сукцината натрия) в дозе 100 мг в сутки (25 мг каждые 6 часов); при плохом самочувствии, низком артериальном давлении дозу можно увеличить на 50–100%.Далее, при отсутствии осложнений — постепенно (уменьшение на 30% в сутки) вернуться к базисной заместительной терапии в течение 5–7 дней. В зависимости от возможности энтерального питания, переход на таблетированную терапию.Исследование уровней калия, натрия, глюкозы в крови
Болезни, которые требуют интенсивной терапии (реанимационные мероприятия), например, септический шок	Непрерывное введение раствора гидрокортизона (в виде гидрокортизона сукцината натрия) 200 мг в сутки (или по 50 мг каждые 6 ч внутривенно или внутримышечно); далее при улучшении состояния пациента ежедневное снижение дозы раствора гидрокортизона на 30% с последующим переводом на таблетированный гидрокортизон в комбинации с флудрокортизоном
Тяжелые неинфекционные заболевания: инфаркт миокарда, панкреатит, тяжелая травма	Раствор гидрокортизона (в виде гидрокортизона сукцината натрия) 150 мг в сутки внутривенно (например, 50 мг каждые 8 ч) до нормализации состояния; далее при улучшении состояния пациента ежедневное снижение дозы раствора гидрокортизона на 30% с последующим переводом на таблетированный гидрокортизон в комбинации с флудрокортизоном
Аддисонический криз	Внутривенное болюсное введение раствора гидрокортизона (в виде гидрокортизона сукцината натрия) в дозе 100 мг, далее непрерывное введение 200 мг в сутки; на следующий день — 200 мг в сутки; далее, при улучшении состояния пациента, ежедневное снижение дозы раствора гидрокортизона на 30% (150 мг, 100 мг, 75 мг, далее 50 мг) с последующим переводом на таблетированный гидрокортизон в дозе 30 мг в комбинации с флудрокортизоном в начальной дозе 100 мкг;внутривенное введение 0,9%-ного раствора натрия хлорида 1000 мл в течение первого часа или 5%-ного раствора декстрозы в 0,9%-ном растворе натрия хлорида, далее непрерывное внутривенное введение 0,9%-ного раствора натрия хлорида при необходимости;контроль гемодинамики;ежедневное (при наличии показаний — чаще) исследование уровней натрия, калия и глюкозы в крови.Эквивалентные дозы раствора преднизолона составляют: в первые сутки — 75 мг, на следующие сутки — 50 мг, далее — 30 мг, 20 мг, 10 мг. Флудрокортизон в дозе 100 мкг — после достижения дозы преднизолона менее 12,5 мг.Эквивалентные дозы раствора дексаметазона составляют: в первые сутки — 12 мг, на следующие сутки — 8 мг, далее — 6 мг, 4 мг, 3 мг, 2 мг, 1 мг. Флудрокортизон в дозе 100 мкг — с первых суток лечения

Несмотря на оптимальную заместительную терапию, значительное количество пациентов продолжают предъявлять объективные и субъективные жалобы: симптомы передозировки и декомпенсации, снижение работоспособности и физической активности, особенно у женщин, ухудшение общего состояния здоровья, метаболические и сердечно-сосудистые осложнения, включая артериальную гипертензию. Новые препараты с модифицированным высвобождением гидрокортизона в некоторой степени имитируют циркадный ритм секреции эндогенного кортизола [54]. Препараты с двойным и медленным (отсроченным) высвобождением максимально приблизились к этому.

Так, гидрокортизон двойного высвобождения одобрен для применения в ряде европейских стран с конца 2012 г. Препарат выпускается в дозах 5 и 20 мг и состоит из наружной оболочки, содержащей гидрокортизон быстрого высвобождения, и внутреннего ядра с гидрокортизоном замедленного высвобождения. Препарат принимается однократно утром [55]. В когорте пациентов с ВДКН, которые были переведены на данный препарат, отмечено улучшение липидного профиля, однако, несмотря на увеличение эквивалентной дозы гидрокортизона, контроль заболевания ухудшился (зафиксирована тенденция к повышению уровней 17ОНР и андростендиона) [56].

Гидрокортизон медленного высвобождения состоит из внутреннего ядра с гидрокортизоном, покрытым рН-чувствительным слоем с замедленным высвобождением. В результате приема препарата пиковый выброс кортизола отмечается в отсроченном периоде и менее выражен. На основании фармакокинетической модели, физиологический уровень кортизола может быть достигнут при приеме 15 или 20 мг препарата в 23:00 и меньшей дозы 10 мг в 07:00 [51]. Согласно данным клинического исследования 2 фазы, профиль кортизола при приеме гидрокортизона медленного высвобождения (в начальной дозе 10 мг в 07:00 и 20 мг в 23:00 с дальнейшей титрацией) у пациентов с ВДКН был приближен к физиологическому. Более того, на фоне данной терапии отмечено уменьшение потребности в суточной дозе гидрокортизона. В исследовании препарат назначался пациентам в капсулах по 5, 10 или 20 мг [54].

Не менее важным аспектом терапии препаратом замедленного высвобождения является более эффективное снижение пика выброса АКТГ и надпочечниковых андрогенов. Терапия данным препаратом приводила к нормализации уровней 17ОНР и андростендиона [54], а также к значимому снижению уровня их метаболитов в моче [57], несмотря на снижение эквивалентной дозы.

В 2019 г. завершилось многоцентровое открытое рандомизированное исследование 3‑й фазы данного препарата (включено 122 пациента с ВДКН, получавших гидрокортизон медленного высвобождения или стандартную терапию различными препаратами глюкокортикоидов в течение 6 месяцев). Примечательно, что на фоне стандартной терапии в 4,92% случаев развился аддисонический криз, тогда как на фоне приема препарата отсроченного высвобождения данное состояние не развилось ни у одного пациента [58].

## Заместительная терапия минералокортикоидами

Сольтеряющая форма ВДКН подразумевает обязательное комбинированное назначение препаратов глюко- и минералокортикоидов. Типичной ошибкой при проведении заместительной терапии сольтеряющей формы ВДКН является назначение монотерапии глюкокортикоидами, в лучшем случае гидрокортизоном, но чаще всего преднизолоном. В этой ситуации увеличение дозы препарата состояние больных не нормализует, а приводит в ряде случаев к развитию экзогенного синдрома Кушинга [59].

Современная заместительная терапия ВДКН минералокортикоидами подразумевает использование только одного препарата — флудрокортизона. Флудрокортизон обычно назначается утром, так как уровень эндогенного альдостерона в норме является самым высоким в это время, после перемены положения тела из горизонтального в вертикальное. Период полувыведения флудрокортизона составляет 18–36 часов, что позволяет назначать препарат однократно. Стартовая доза обычно составляет 50–100 мкг. Поддерживающая суточная доза для взрослых и подростков обычно составляет 50–200 мкг и зависит от потребления/потери жидкости и электролитов. У детей чувствительность к МК ниже, поэтому требуются более высокие дозы флудрокортизона по сравнению теми, что рассчитаны на взрослых. При чрезмерном потоотделении, например, в условиях жаркого климата, необходимо временное увеличение дозы на 50–100% и увеличение потребления соленых продуктов. На фоне терапии преднизолоном и тем более дексаметазоном, который не обладает минералокортикоидной активностью, может потребоваться доза флудрокортизона больше, чем на фоне лечения гидрокортизоном. Необходимо учитывать, что лакрица и грейпфрутовый сок усиливают минералокортикоидный эффект гидрокортизона и должны исключаться из употребления, а фенитоин усиливает метаболизм флудрокортизона, в связи с чем дозу препарата приходится увеличивать [60].

Компенсация минералокортикоидной недостаточности оценивается по клинико-лабораторным показателям:

После начала терапии флудрокортизоном у некоторых пациентов развиваются легкая отечность. Это явление не следует воспринимать как основание для отмены препарата, спустя несколько дней (максимум неделю) оно обычно саморазрешается.

Благодаря мощным минералокортикоидным и сользадерживающим свойствам флудрокортизон используется также в лечении идиопатической гипотонической болезни и ортостатической артериальной гипотонии.

Передозировка минералокортикоидными препаратами приводит к развитию отеков, повышению артериального давления и гипокалиемии (табл. 6).

**Table:** Таблица 6. Клинические проявления гипокалиемии

Нарушения	Проявление
Нервно-мышечные	слабость, запоры, депрессия, обморочное состояние
Кардиологические	аритмии, усиление токсичности сердечных гликозидов, изменение ЭКГ (снижение ST сегмента, уменьшение/инверсия зубца Т, удлинение P-R интервала, увеличение зубца U)
Почечные	ухудшение концентрирующей способности, приводящее к полиурии и полидипсии, распад мышечной ткани сопровождается повышением креатинина в моче
Метаболизма	алкалоз

В тяжелых случаях передозировки рекомендуется временно прекратить прием флудрокортизона и увеличить дозу ГК, проводить дегидратацию и борьбу с гипокалиемией, которая купируется введением раствора хлорида калия на 5%-ном растворе глюкозы. При отеке мозга с осторожностью применяют мочегонные средства.

У пациентов с сольтеряющей формой ВДКН и артериальной гипертензией необходимо оценивать адекватность и минерало-, и глюкокортикоидной терапии, так как передозировка любого из препаратов может повышать артериальное давление. При артериальной гипертензии необходимо уменьшить дозу флудрокортизона, а если артериальное давление остается повышенным и нет отеков, необходимо назначить гипотензивную терапию, а лечение флудрокортизоном продолжить. У пациентов с ВДКН и артериальной гипертензией преимущественно должны назначаться гипотензивные препараты, купирующие вазоконстрикторный эффект ангиотензина II: блокаторы рецептора ангиотензина II или блокаторы ангиотензинпревращающего фермента. Препаратами второго ряда являются дигидропиридиновые блокаторы кальциевых каналов. Мочегонные препараты назначаются в исключительных случаях, а блокаторы минералокортикоидных рецепторов противопоказаны [60].

## Лечение неклассической формы ВДКН

## Новое в терапии ВДКН

Новым подходом к терапии ВДКН является блокада гипоталамо-гипофизарно-надпочечниковой системы, в частности АКТГ, как главного стимулятора коры надпочечников. Такая терапия по-прежнему требует назначения ГК для восполнения дефицита кортизола. Однако данная схема «блокируй-замещай» благодаря более эффективной супрессии гипоталамо-гипофизарно-надпочечниковой системы позволяет уменьшить дозу ГК [51].

В этой связи представляется перспективным применение антагонистов рецепторов кортикотропин-рилизинг-гормона, который является основным регулятором синтеза и секреции АКТГ. Нарушение секреции АКТГ позволяет, соответственно, снизить секрецию надпочечниковых андрогенов без назначения супрафизиологических доз ГК [51].

Так, согласно результатам проведенных исследований, терапия антагонистом рецепторов к кортикотропин-рилизинг-гормону 1 типа тильдасерфонтом позволила снизить уровни АКТГ, андростендиона и 17ОНР у пациентов с неудовлетворительным контролем заболевания, но не влияла на уровни андростендиона и 17ОНР у пациентов с удовлетворительным контролем [61].

Терапия другим антагонистом рецепторов к кортикотропин-рилизинг-гормону 1 типа, кринсерфонтом, также приводила к дозозависимому снижению уровня АКТГ (медиана изменения показателя 54–66%) и 17ОНР (медиана изменения 21–64%). Также зафиксировано снижение уровня тестостерона у женщин и соотношения андростендион/тестостерон у мужчин [62].

В рандомизированном двойном слепом плацебо-контролируемом исследовании [63] показано, что применение кринсерфонта у пациентов с ВДКН позволило снизить дозу ГК с супрафизиологической до физиологической без увеличения частоты аддисонических кризов. Согласно данным систематического обзора и метаанализа [64], значимой разницы в выраженности снижения АКТГ у пациентов, получающих кринсерфонт и тильдасерфонт, не выявлено. Однако применение первого препарата позволило достичь более выраженного снижения 17ОНР и андростендиона.

## Перспективы генетической и клеточной терапии ВДКН

Современные исследования в области терапии дефицита 21-гидроксилазы развиваются по двум основным направлениям: генная терапия, направленная на компенсацию дефектного гена CYP21A2, и клеточная терапия, ставящая целью создание и трансплантацию функциональных стероидогенных клеток.

Генная терапия концентрируется на стратегии заместительной экспрессии CYP21A2 с использованием аденоассоциированных векторов (AAV). Проходят клинические исследования I/II фазы препарата BBP-631 (AAV5-CYP21A2), в рамках которых будет оценена безопасность и переносимость терапии, возможность снижения дозировок или отказа пациентов с классической формой ВДКН от заместительной терапии глюко- и минералокортикоидами [65].

Успешное использование AAV ранее было продемонстрировано как на клеточных культурах пациентов, так и на животных моделях. Особый интерес представляет концепция «гепато-адренальной кооперации», согласно которой экспрессия CYP21A2 в клетках печени способна частично компенсировать нарушенный стероидогенез в надпочечниках [66][67]. Несмотря на обнадеживающие результаты, перед широким внедрением метода предстоит решить ряд ключевых задач: обеспечить эффективную доставку вектора именно в кору надпочечников, добиться долговременной стабильности экспрессии гена, а также минимизировать потенциальный иммунный ответ на капсид вектора или трансген. Для адекватной оценки этих параметров критически важным является развитие релевантных доклинических моделей, адекватно воспроизводящих заболевание [68].

Клеточные подходы предлагают альтернативное решение, нацеленное на восстановление физиологического регуляторного узла, секретирующего кортизол. В рамках этого направления успешно получены индуцированные стероидогенные клетки человека (hiSC) как из соматических клеток пациентов, так и из линий плюрипотентных клеток. hiSC клетки демонстрируют экспрессию ключевых стероидогенных ферментов и способны синтезировать кортикостероиды in vitro. Текущие исследования сосредоточены на оптимизации протоколов дифференцировки, поиске идеального клеточного источника и разработке методов трансплантации, включая использование защищающих капсул для предотвращения отторжения [69][70].

Параллельно исследуются и более инновационные стратегии, такие как доставка генетических конструкций с помощью липидных наночастиц и редактирование генома in vivo или ex vivo. Однако применительно к ВДКН эти подходы пока находятся на ранних стадиях разработки и сталкиваются с проблемами эффективного таргетинга коры надпочечников и обеспечения долгосрочной безопасности [71].

Таким образом, современная парадигма лечения ВДКН постепенно смещается от пожизненной гормональной терапии глюко- и минералокортикоидами в сторону методов, направленных на устранение генетической причины заболевания. Несмотря на существующие вызовы, связанные с безопасностью, таргетной доставкой и стабильностью эффекта, активное развитие доклинических моделей и регенеративной медицины формирует прочный фундамент для создания принципиально новых методов лечения ВДКН.

## ЗАКЛЮЧЕНИЕ

Современный этап изучения ВДКН характеризуется значительным углублением представлений о молекулярно-генетических основах заболевания. Достижения в области генетики, прежде всего картирование и клонирование гена CYP21A2, объяснение механизмов конверсии и крупных перестроек в RCCX-локусе, позволили перейти к созданию точных систем диагностики, включая неонатальный скрининг и пренатальную диагностику. Тем не менее ВДКН остается трудно корректируемой патологией, при которой пожизненная гормональная терапия зачастую не позволяет достичь оптимального метаболического контроля. Хроническая гиперпродукция андрогенов, эпизоды ятрогенного гиперкортицизма на фоне неоптимальной медикаментозной терапии, а также постоянный риск развития аддисонических кризов при интеркуррентных заболеваниях значимо снижают качество жизни пациентов и предопределяют высокую частоту отдаленных осложнений. Ближайшие перспективы связаны не с революционной заменой существующей терапии, а с ее оптимизацией. К таковой можно отнести внедрение препаратов гидрокортизона с модифицированным высвобождением, более адекватно воспроизводящих физиологический циркадный ритм секреции кортизола, а также клиническое апробирование новых схем лечения, в частности комбинации заместительных доз глюкокортикоидов с антагонистами кортикотропин-рилизинг-гормона. Перспективы дальнейших исследований связаны с развитием методов генной и клеточной терапии, направленных на устранение первичной причины заболевания. Таким образом, несмотря на достигнутый прогресс, ВДКН продолжает оставаться серьезной медико-социальной проблемой, решение которой требует дальнейших комплексных исследований, направленных на совершенствование методов контроля заболевания и улучшение долгосрочных клинических исходов.

## ДОПОЛНИТЕЛЬНАЯ ИНФОРМАЦИЯ

Источники финансирования. Работа выполнена по инициативе авторов без привлечения финансирования.

Конфликт интересов. Авторы декларируют отсутствие явных и потенциальных конфликтов интересов, связанных с содержанием настоящей статьи.

Участие авторов. Все авторы одобрили финальную версию статьи перед публикацией, выразили согласие нести ответственность за все аспекты работы, подразумевающую надлежащее изучение и решение вопросов, связанных с точностью или добросовестностью любой части работы.
